# From farm to flask: stable genotypes and shifting microbiomes in the ecological dynamics of *Balantioides coli* from a One Health perspective

**DOI:** 10.1186/s13071-026-07513-y

**Published:** 2026-07-07

**Authors:** Camila Souza Carvalho Class, Pedro Mendes de Souza, Renan de Souza Ferreira, Ingrid da Silva Reis, Laís Lisboa Corrêa, Breno Torres da Silva, Gabriella Ribeiro Vaz da Costa, Fabiana Batalha Knackfuss, Roberto Júnio Pedroso Dias, Alynne da Silva Barbosa

**Affiliations:** 1 Department of Microbiology and Parasitology, Biomedical Institute, Fluminense Federal University (UFF), Niterói, Rio de Janeiro Brazil; 2 Laboratory of Protozoology, Institute of Biological Sciences, Federal University of Juiz de Fora (UFJF), Juiz de Fora, Minas Gerais Brazil; 3 Faculty of Veterinary Medicine, Centro Universitário Serra dos Órgãos (UNIFESO), Teresópolis, Rio de Janeiro Brazil; 4 Laboratory of Statistics, UNIGRANRIO (AFYA), Duque de Caxias, Rio de Janeiro Brazil

**Keywords:** *Balantioides coli*, Pigs, Gut microbiota, Metabarcoding, Genetic diversity

## Abstract

**Background:**

*Balantioides coli* is a zoonotic intestinal protozoan distributed at the human–animal interface, with pigs recognized as its principal hosts and reservoirs. Despite its broad occurrence, gaps remain regarding its genetic diversity and its interactions with associated microbial communities. This study investigated the genetic and phylogenetic diversity of *B. coli* and the impact of in vitro culture on the structure of these communities using Sanger and next-generation sequencing (NGS) in xenic isolates and their original fecal samples.

**Methods:**

A total of 39 fecal samples from pigs were collected between 2023 and 2024 from 15 farms located in the states of Rio de Janeiro and Minas Gerais, Brazil. Samples were subjected to direct microscopy, xenic culture, Sanger sequencing of the ITS1–5.8SrDNA–ITS2 region, and NGS targeting the 18S and 16S rRNA regions.

**Results:**

Direct examination revealed a higher frequency of trophozoites (64.1%) compared with cysts (48.7%). In vitro isolation was successful from fecal samples obtained from pigs at all farms. Molecular ITS analysis confirmed the presence of *B. coli* in both isolates and fecal samples, with predominance of the B0 variant. Metabarcoding revealed that the abundance and genetic diversity of other eukaryotes did not differ significantly between feces and cultures (p > 0.05), although fecal samples exhibited greater taxonomic richness, particularly *Blastocystis* spp. and *Entamoeba* spp., including *E. suis*, *E. polecki*, *E. moshkovskii*, and *E. hartmanni*. In contrast, prokaryotic communities showed significant differences (p ≤ 0.05). In correspondence analyses, the eukaryotic dataset showed a diffuse distribution of *B. coli* ASVs, without marked associations with other eukaryotic taxa. In contrast, the prokaryotic dataset showed more structured associations, with a large cluster of *B. coli* ASVs grouped near bacterial genera such as *Bacteroides*, *Pseudomonas*, and *Escherichia–Shigella*.

**Conclusions:**

*Balantioides coli* remained genetically stable after in vitro establishment, with predominance of the B0 variant in both fecal samples and xenic cultures. Xenic culture preserved part of the eukaryotic diversity but significantly restructured the prokaryotic community, selecting bacterial taxa potentially linked to parasite persistence under in vitro conditions. These findings indicate that *B. coli* persists within a complex intestinal microbial consortium rather than as an isolated protozoan.

**Graphical Abstract:**

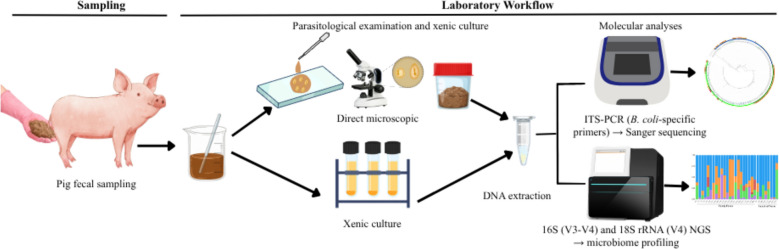

**Supplementary Information:**

The online version contains supplementary material available at 10.1186/s13071-026-07513-y.

## Background

*Balantioides coli * is a neglected zoonotic intestinal protozoan widely prevalent at the human–animal interface, specifically within pig farms, where elevated infection rates likely drive continuous environmental transmission [1, 2]. Pigs and non-human primates are considered its main natural reservoirs, and it remains the only known ciliate capable of infecting humans, preferentially colonizing the large intestine [2–4]. In humans and non-human primates, infection may cause severe disease, including dysentery and extraintestinal involvement [4–6]. Transmission occurs predominantly via the fecal–oral route, through ingestion of water or food contaminated with environmentally resistant cysts, highlighting its One Health relevance [2–4].

Within pig production systems, infection dynamics may be influenced by differences in husbandry practices and sanitary management. Industrial farms generally adopt stricter biosecurity measures, whereas family-based systems often are characterized by less standardized practices and greater environmental exposure, which may affect parasite circulation and associated microbial communities [7–11].

Although detection has traditionally relied on microscopic detection of trophozoites and cysts, these methods are limited by low sensitivity and poor taxonomic resolution [12–17]. Consequently, molecular approaches based on PCR and sequencing of ribosomal markers, particularly the ITS regions, have become essential for accurate identification and genetic characterization [16, 18]. Such investigations have uncovered significant intraspecific diversity within humans and various animal hosts, comprising variants A0, A1, A2, B0, and B1 as well [18–20].

In parallel, in vitro xenic culture, in which the parasite is grown in the presence of an undefined microbiota, has become an important tool for increasing parasite biomass and enabling downstream morphological and molecular investigations, with Jones’ modified Pavlova medium remaining one of the most widely used systems [3, 17, 21]. However, because *B. coli* is commonly maintained in association with other microorganisms, microbial interactions may play a critical role in parasite survival and successes in these cultures, although these relationships remain poorly understood.

Recent advances in next-generation sequencing (NGS) and metabarcoding targeting the 18S and 16S rRNA genes have enabled the simultaneous characterization of eukaryotic and prokaryotic communities associated with the parasite [22–24]. Nevertheless, comparative studies between fecal samples and corresponding xenic cultures remain scarce, limiting our understanding of whether in vitro culture preserves the genetic diversity of *B. coli* and its associated microbiota or promotes selective shifts.

Therefore, this study aimed to compare the genetic diversity of *B. coli* in pig feces and corresponding xenic cultures, assess culture-driven microbial shifts and co-occurrence patterns, and evaluate these dynamics across different pig production systems.

## Methods

### Sampling and *Balantioides coli* isolation

Fecal samples were collected between July 2023 and July 2024 from pig farms located in the states of Rio de Janeiro and Minas Gerais, Brazil. A total of 15 farms were included, comprising four industrial and eleven family-based production systems, anonymized as farms A–O (Additional file 1: Table S1). Samples were collected directly from the rectal ampulla using lubricated gloves [1].

Immediately after collection, samples were diluted in sterile phosphate-buffered saline and microscopically examined for Ciliophora trophozoites and cysts. For each municipality, two fecal samples were selected for in vitro isolation. In industrial farms with herds larger than 100 animals, additional samples were included to improve herd representativeness (Additional file 1: Table S1).

Priority was given to samples with the highest number of ciliates-compatible forms, whereas in farms with no positive samples, two fecal samples were randomly selected and inoculated blindly into the culture medium. The original fecal sample corresponding to each isolate was simultaneously processed for DNA extraction.

For in vitro isolation, fecal material was inoculated into Pavlova medium [25], modified according to Jones [26] and supplemented with fetal bovine serum. Inoculation procedures and isolate evaluation followed our previous protocols [20, 21]. An antibiotic solution containing penicillin (final concentration: 10,000 IU/mL) and streptomycin sulfate (final concentration: 10,000 µg/mL) was added to the culture medium to control excessive bacterial growth.

The inoculum was prepared from fresh feces diluted in sterile buffered saline solution. Samples were inoculated in duplicate into tubes containing 8 mL of fresh culture medium supplemented with 60 µL of sterile rice starch suspension. Approximately 180 µL of fecal suspension was added to each tube using a sterile Pasteur pipette. Culture tubes were maintained hermetically sealed throughout the incubation period, without active gas exchange or controlled atmospheric supplementation. No anaerobic jars or specific gas mixtures were used. Cultures were incubated in a bacteriological incubator at 36 °C based on the aerotolerant characteristics of *B. coli*. Under these conditions, oxygen availability was likely gradually reduced by microbial and protozoan metabolic activity within the closed culture system, generating a microenvironment favorable for trophozoite maintenance and proliferation. All cultures were processed simultaneously under identical experimental conditions to minimize technical variability.

Cultures were examined after 24 h of incubation by microscopic observation of a sediment aliquot under 100 × magnification (Primo Star, Zeiss®). Culture viability was determined by the presence of motile trophozoites. Positive cultures were subcultured into fresh medium, whereas negative cultures were reincubated and re-evaluated every 24 h for up to 72 h. Cultures remaining negative after this period were discarded. Positive isolation was defined by the detection of motile trophozoites during the initial verification stage.

Successfully isolated parasites were maintained and subcultured in fresh medium every 24 h until the fifth day of incubation. At this time point, material from two culture tubes of each isolate was subjected to DNA extraction. Routine subculturing was subsequently continued according to the previously established protocol [20, 21] for further analyses and evaluation of long-term maintenance under xenic conditions.

### DNA extraction, polymerase chain reaction, and Sanger sequencing

DNA was extracted from both cultured isolates and the corresponding original fecal samples using the QIAamp Fast DNA Stool Mini Kit (Qiagen®), following the manufacturer’s instructions with previously described modifications [19].

All extracted DNA samples were subjected to PCR using Platinum Hot Start Master Mix (Invitrogen®) and the primers B58D and B58RC, targeting the ITS1–5.8SrDNA–ITS2 region of Ciliophora and generating amplicons of approximately 500 bp, as previously described by Ponce-Gordo et al. [27]. Each reaction contained 5 μL of template DNA. PCR products were visualized on a 1.5% agarose gel stained with GelRed (Biotium^®^) and bromophenol blue (LGC^®^), then purified using ExoSAP-IT (Invitrogen^®^), and submitted for Sanger sequencing on an ABI 3730xL automated sequencer (Additional file 1: Table S1). Negative controls (ultrapure water) and positive controls consisting of *B. coli* culture material from our research group were included in all extraction and PCR runs.

Sequences were edited in ChromasPro® v.1.7.5 and combined with reference sequences retrieved from GenBank, including *B. coli* sequences from domestic pigs from different countries representing well-defined genetic variants (326–600 bp). Sequences of *Balantidium entozoon*, *Buxtonella sulcata*, and *Buxtonella-like* organisms were included as outgroups, while sequences with ambiguous variant annotations were excluded.

The final dataset (n = 288) was aligned using MAFFT [28] and refined with GBLOCKS [29] to remove poorly aligned regions, followed by visual inspection in SeaView v.5.0.5. Maximum likelihood phylogenetic reconstruction was performed in the IQ-TREE software [30] with 1000 bootstrap pseudoreplicates using the best-fit substitution model automatically selected by the software.

### Library preparation and Illumina sequencing

DNA from 15 pig fecal samples and corresponding cultured isolates was subjected to metabarcoding (Additional file 1: Table S1). A total of 30 paired samples were processed, comprising 15 raw fecal samples and 15 corresponding culture isolates obtained from the same properties. Due to the high financial costs associated with large-scale sequencing analyses, one representative sample per property was selected for cultivation and subsequent metabarcoding analysis.

Sequencing was performed by Genone^®^ (Illumina HiSeq 2500) after PCR amplification of the 16S rRNA (V3–V4; 341F/806Rb) and 18S rRNA (V4; TAReuk454FWD1/TAReukREV3) regions [31, 32].

Following initial quality control by the sequencing provider, all downstream analyses were performed in QIIME 2 [33]. Adapters were removed and paired-end reads were demultiplexed using q2-cutadapt [34] and q2-demux, respectively. Reads were then merged, denoised, and dereplicated using q2-dada2 [35]. Sequences with < 20 bp overlap, reads shorter than 225 bp, and chimeric sequences were excluded. The resulting amplicon sequence variants (ASVs) were taxonomically assigned using the feature-classifier plugin with the sklearn module trained on the PR2 database [36].

### Statistical and bioinformatic analyses

Nucleotide sequences of *B. coli* obtained by Sanger sequencing were phylogenetically compared with reference sequences from domestic pigs available in GenBank, considering the country of origin and genetic variant classification. Relative abundance analyses and Venn diagrams were used to explore the taxonomic composition of eukaryotic and prokaryotic communities, as well as potentially pathogenic parasites detected in pig fecal samples and *B. coli* xenic cultures. These plots were generated in Python using Matplotlib [37], Plotnine [38], and Pandas [39].

Microbiota diversity and dissimilarity between fecal samples and xenic cultures were assessed by principal coordinates analysis (PCoA) based on Bray–Curtis dissimilarity and Weighted UniFrac distance, with significance tested by PERMANOVA using 999 permutations (p ≤ 0.05) in the q2-diversity plugin of QIIME 2 [33].

Potential interactions between *B. coli* and other prokaryotic and eukaryotic genera were investigated through Correspondence Analysis (CA) in Python. For this analysis, ASV abundance data for both prokaryotes and non-balantidiid eukaryotes were agglomerated at the genus level. To ensure robust statistical patterns, only genera with a total cumulative abundance exceeding 1000 reads across all samples were retained for downstream analysis. Data processing and visualization were performed using Pandas [39], SciPy [40], Statsmodels [41], NumPy [42], and Matplotlib [37].

The geographic distribution of sampling sites was mapped in QGIS v.3.34.14 “Prizren” [43]. Pie charts representing the composition of prokaryotic and eukaryotic communities in fecal and culture samples were generated in Python and overlaid onto the geographic coordinates of each farm.

## Results

### Direct examination, in vitro culture, and molecular characterization of *Balantioides coli* genetic variants

In direct examination, trophozoites and cysts were observed with variable frequencies among samples. Trophozoites were detected in 25 of the 39 samples analyzed, with counts ranging from 1 to 180 forms per slide. Cyst detections were less frequent (19/39), with counts ranging from 1 to 57 structures per slide (Table 1).

**Table 1:** Direct examination, culture, and molecular results of *Balantioides coli*-positive pig fecal samples from Brazil

City (farm)	Sample ID	Direct examination		Culture	PCR and Sanger	
		Trophozoites (count)	Cysts (count)	Maintenance (days)	Fecal variant	Culture variant
Maricá (A)	71	2	0	111	*B. coli* **B0** (LC899043)	*B. coli* **B0** (LC899042)
Maricá (B)	77	6	57	11	*B. coli* **B0** (LC899045)	*B. coli* **B0** (LC899044)
Cachoeiras de Macacu (C)	82	2	0	55	*B. coli* **A0** (LC899047)	*B. coli* **A0** (LC899046)
Cachoeiras de Macacu (D)	119	3	6	16	*B. coli* **B0** (LC899049)	*B. coli* **B0** (LC899048)
Silva Jardim (E)	129	3	11	19	*B. coli* **B0** (LC899051)	*B. coli* **B0** (LC899050)
Silva Jardim (E)	150	38	0	16	*B. coli* **B0** (LC899053)	*B. coli* **B0** (LC899052)
Rio Bonito (F)	157	4	0	12	*B. coli* **A0** (LC899055)	*B. coli* **B0** (LC899054)
Rio Bonito (G)	158	0	5	12	*B. coli* **B0** (LC899057)	*B. coli* **B0** (LC899056)
Itaboraí (H)	177	0	0	380	*B. coli* **B0** (LC899059)	*B. coli* **B0** (LC899058)
Itaboraí (H)	181	10	0	12	*B. coli* **B0** (LC899061)	*B. coli* **B0** (LC899060)
Saquarema (I)	251	1	1	23	*B. coli* **B0** (LC899063)	*B. coli* **B0** (LC899062)
Saquarema (I)	257	0	8	32	*B. coli* **B0** (LC899065)	*B. coli* **B0** (LC899064)
Casimiro de Abreu (J)	717	2	0	33	*B. coli* **B0** (LC899086)	*B. coli* **B0** (LC899085)
Casimiro de Abreu (J)	719	0	50	15	*B. coli* **B0** (LC899088)	*B. coli* **B0** (LC899087)
Tanguá (K)	745	0	5	17	*B. coli* **B0** (LC899090)	*B. coli* **B0** (LC899089)
Tanguá (K)	760	0	2	0	*B. coli* **B0** (LC899091)	NI
Pinheiral (L)	18	28	0	31	*B. coli* **A0** (LC899039)	*B. coli* **B0** (LC899038)
Pinheiral (L)	27	21	0	73	*B. coli* **B0** (LC899041)	*B. coli* **A0** (LC899040)
Nova Friburgo (M)	283	0	2	33	*B. coli* **B0** (LC899067)	*B. coli* **B0** (LC899066)
Nova Friburgo (M)	284	0	1	15	*B. coli* **B0** (LC899069)	*B. coli* **B0** (LC899068)
Nova Friburgo (M)	379	0	1	0	*B. coli* **B0** (LC899070)	NI
Nova Friburgo (M)	383	0	3	13	*B. coli* **B0** (LC899072)	*B. coli* **B0** (LC899071)
Nova Friburgo (M)	418	0	3	20	*B. coli* **A0** (LC899076)	*B. coli* **B0** (LC899075)
Nova Friburgo (M)	458	18	0	24	*B. coli* **A0** (LC899078)	*B. coli* **B0** (LC899077)
Nova Friburgo (M)	462	26	0	33	*B. coli* **A0** (LC899080)	*B. coli* **B0** (LC899079)
Nova Friburgo (M)	565	30	0	13	*B. coli* **B0** (LC899082)	*B. coli* **B0** (LC899081)
Nova Friburgo (M)	586	30	0	13	*B. coli* **B0** (LC899084)	*B. coli* **B0** (LC899083)
Rio Pomba (N)	803	0	1	30	*B. coli* **A0** (LC899093)	*B. coli* **B0** (LC899092)
Rio Pomba (N)	822	180	0	14	*B. coli* **A0** (LC899095)	*B. coli* **A0** (LC899094)
Rio Pomba (N)	826	60	0	326	*B. coli* **A0** (LC899097)	*B. coli* **A0** (LC899096)
Rio Pomba (N)	849	5	5	16	*B. coli* **B0** (LC899099)	*B. coli* **B0** (LC899098)
Rio Pomba (N)	851	3	0	14	*B. coli* **B0** (LC899101)	*B. coli* **B0** (LC899100)
Rio Pomba (N)	852	30	0	14	*B. coli* **B0** (LC899103)	*B. coli* **B0** (LC899102)
Rio Pomba (N)	862	15	0	14	*B. coli* **B0** (LC899105)	*B. coli* **B0** (LC899104)
Barbacena (O)	927	0	30	8	*B. coli* **B0** (LC899109)	*B. coli* **B0** (LC899108)
Barbacena (O)	930	3	30	13	*B. coli* **B0** (LC899111)	*B. coli* **B0** (LC899110)
Barbacena (O)	959	30	0	6	*B. coli* **B0** (LC899113)	*B. coli* **B0** (LC899112)
Barbacena (O)	961	1	0	6	*B. coli* **B0** (LC899115)	*B. coli* **B0** (LC899114)
Barbacena (O)	970	0	30	8	*B. coli* **B0** (LC899117)	*B. coli* **B0** (LC899116)

A–O indicate the pig farms included in the study; NI, not isolated

In vitro culture was successful for samples originating from all evaluated localities, and parasite isolation was achieved in at least one sample per farm. Of the 39 samples subjected to in vitro isolation, 37 were successfully isolated. Most isolates (27/39) remained viable for 12 to 33 days (Table 1).

Molecular analysis confirmed the presence of *B. coli* in all fecal samples and their corresponding cultured isolates. Variant B0 was predominant, being detected in 76.9% (30/39) of fecal samples and 84.6% (33/39) of cultured isolates and was present in nearly all farms (14/15). Variant A0 occurred at a lower frequency, being identified in 23.0% (9/39) of fecal samples and 10.2% (4/39) of cultures (Table 1). Additionally, one sample that was negative by direct microscopy yielded successful isolation after blind inoculation and was subsequently confirmed as positive by PCR in both the original fecal material and the corresponding culture.

**Fig. 1:**
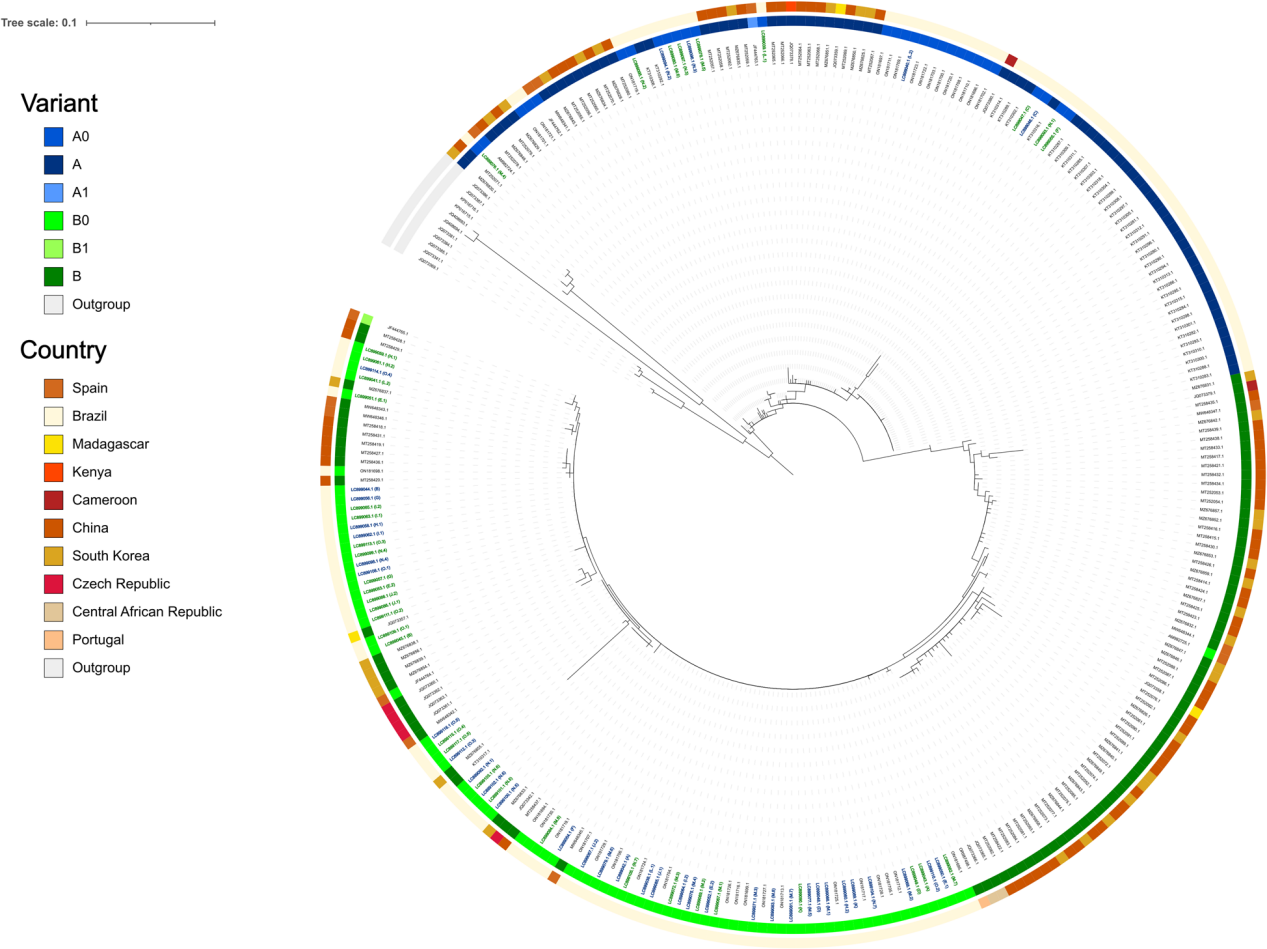
Maximum likelihood phylogeny of ciliates inferred from ITS1–5.8S rDNA–ITS2 sequences. Sequences generated in this study are highlighted in blue for *Balantioides coli* isolates established in vitro and in green for *Balantioides coli*-positive fecal samples. These sequences, obtained from pig fecal samples and in vitro cultures, are shown together with reference sequences representing different countries and genetic variants. Concentric rings indicate: (i) country of origin, outermost ring; and (ii) genetic variant, innermost ring. The scale bar represents 0.1 substitutions per site

Differences between the variant detected directly in fecal samples and that recovered after culture were observed in 17.9% (7/39) of the samples, specifically in feces–culture pairs originating from the farms F, L, M and N (Table 1). Phylogenetic analysis based on the ITS region corroborated the molecular findings, suggesting that variant B lineages **(**B, B0, and B1) constitute a well-supported monophyletic group (Fig. 1). The *B. coli* sequences generated in the present study clustered together with reference sequences deposited in GenBank, showing nucleotide identities ranging from 96.4% to 99.7% for A0 and from 98.3% to 100% for B0 (Fig. 1). For most feces–culture pairs derived from the same host, nucleotide sequences generated from cultured isolates clustered within the same subclade as their corresponding fecal sequences. These clusters were predominantly associated with the B0 variant (Fig. 1).

The genetic distance matrix (Additional file 2: Table S2) further supported this pattern, showing that, in most cases, intra-host genetic distances between feces–culture pairs were close to zero or substantially lower than the distances observed among samples from different farms. However, specific exceptions were confirmed by both phylogenetic reconstruction and the distance matrix, involving the pairs 18F/18C, 27F/27C, 157F/157C, 418F/418C, 458F/458C, 462F/462C, and 803F/803C. In these cases, genetic distances between fecal and culture-derived sequences were higher than those observed for the remaining intra-host pairs and were consistent with a shift between the A0 and B0 variants.

### Unicellular eukaryotic communities associated with *Balantioides coli*: comparison between fecal samples and xenic cultures, and between family-based and industrial farms

**Fig. 2:**
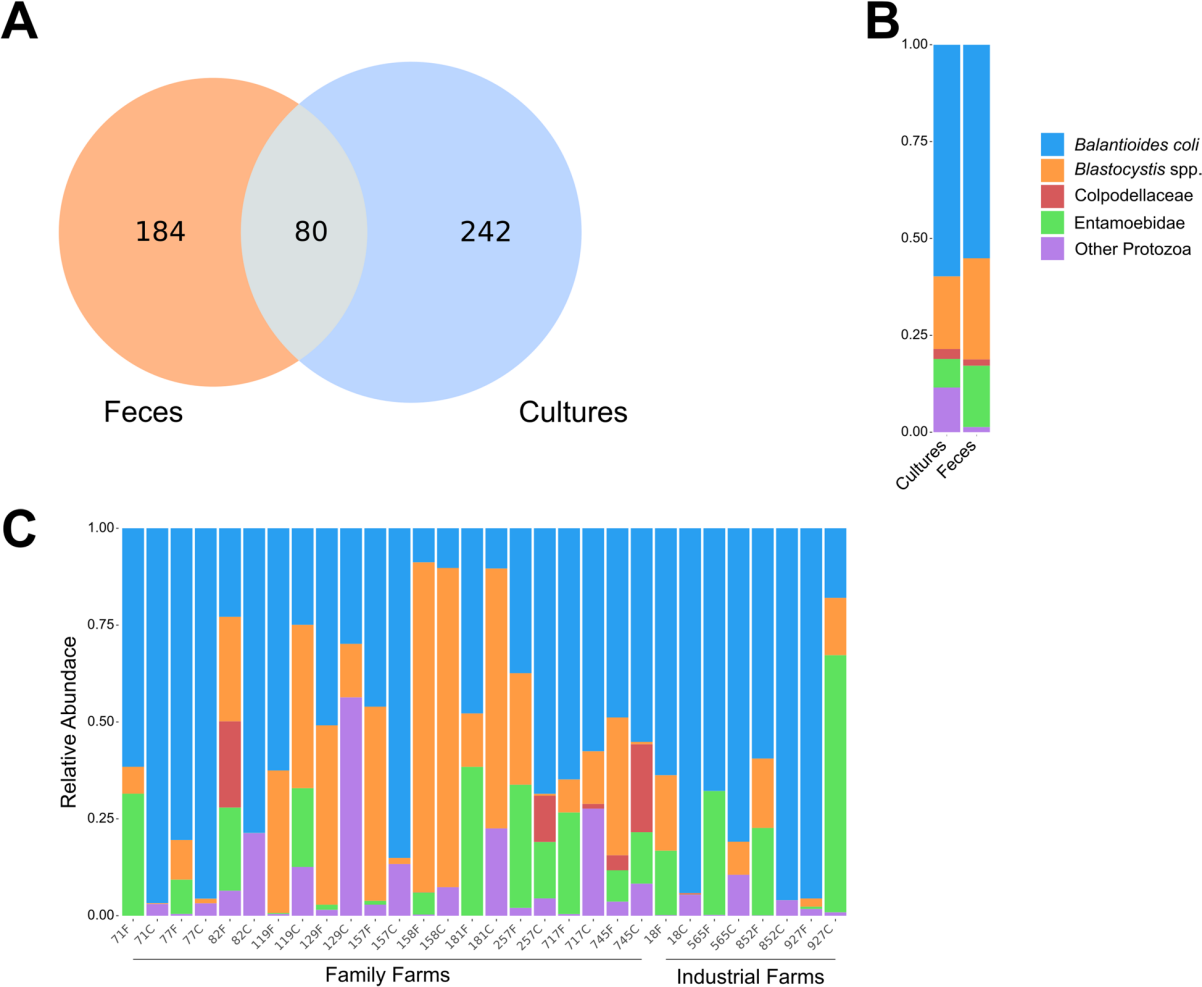
Eukaryotic ASV composition in feces and xenic cultures of *Balantioides coli* across pig production systems. **A** Venn diagram illustrating shared and exclusive unicellular eukaryotic ASVs between pig fecal samples and in vitro xenic cultures of *Balantioides coli*. **B** Overall relative abundance of ASVs corresponding to the main unicellular eukaryotic taxa detected in in vitro xenic cultures of *Balantioides coli* and in the corresponding pig fecal samples from which these isolates were obtained. **C** Relative abundance of ASVs corresponding to the main unicellular eukaryotic taxa identified from xenic in vitro cultures of *Balantioides coli* and in pig fecal samples from family-based and industrial farms, presented individually for each sample

The Venn diagram showed that xenic cultures contained the highest number of exclusive ASVs (n = 242), whereas fecal samples presented 184 distinct eukaryotic ASVs, with 80 ASVs shared between both matrices. Notably, cultures exhibited more than twice the number of shared ASVs relative to their corresponding fecal samples (Fig. 2A).

In xenic cultures, *B. coli* represented the largest proportion of the detected eukaryotic community, followed by *Blastocystis* spp., Entamoebidae, and Colpodellaceae. Fecal samples exhibited a similar overall pattern, maintaining the dominance of *B. coli*, but with a proportionally greater contribution of *Blastocystis* spp. and Entamoebidae compared with in vitro cultured isolates. In addition, a greater diversity and abundance of Entamoebidae ASVs were observed in fecal samples, including the identification of *Entamoeba suis, E. polecki, E. moshkovskii,* and *E. hartmanni*; however, ASVs assigned to the latter species were not detected in culture-derived isolates (Fig. 2B).

Comparison of corresponding feces–culture pairs across farms revealed a consistent pattern of structural shifts in unicellular eukaryotic communities between the two matrices. In cultures, *B. coli* showed marked dominance in most isolates (10/15). In contrast, some feces–culture pairs (6/15) showed predominance of ASVs assigned to *Blastocystis* spp. (119C–119F, 158C–158F, 181C–181F), Entamoebidae (927C–927F), and other eukaryotes (129C–129F) (Fig. 2C).

**Fig. 3:**
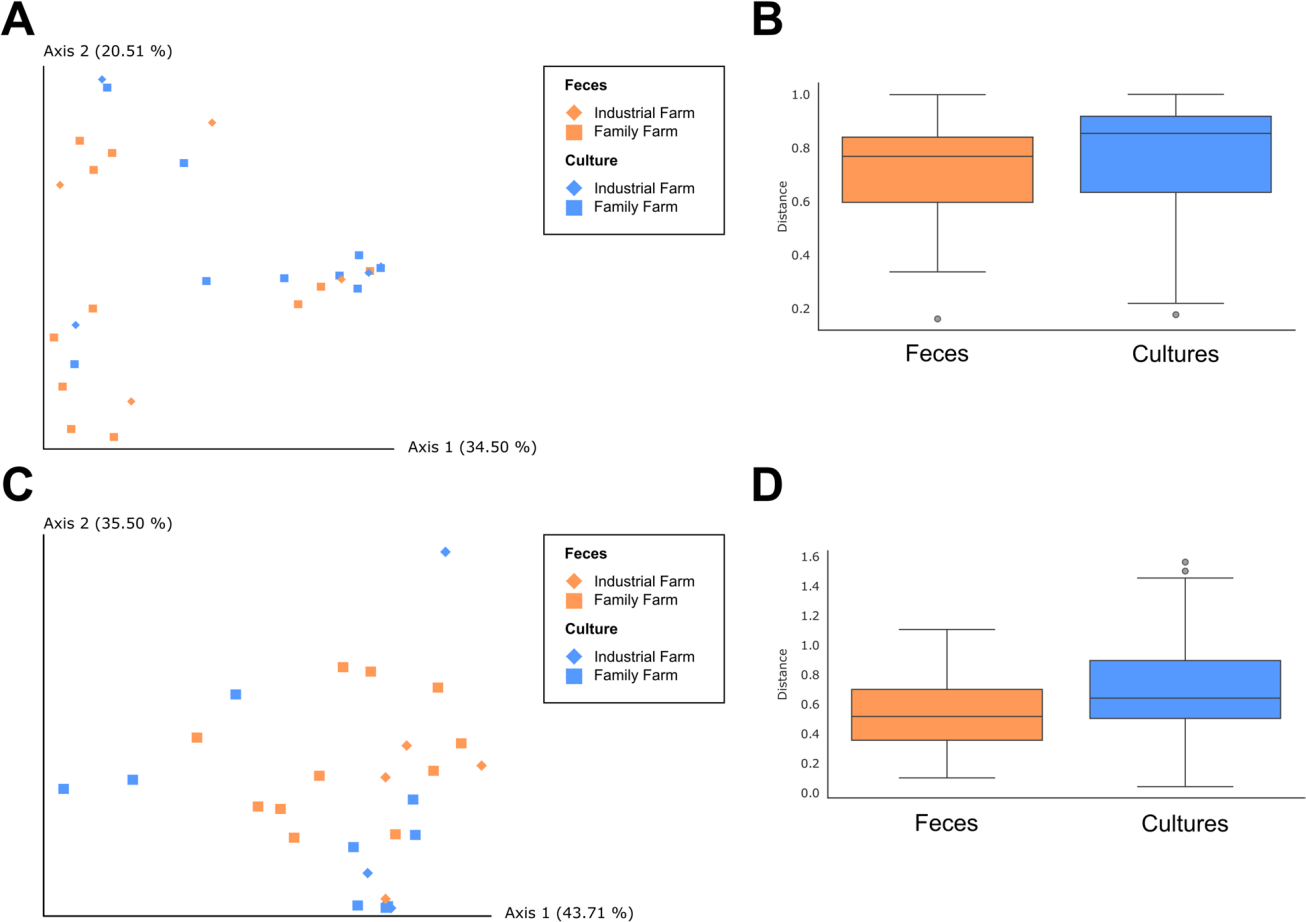
Beta diversity of unicellular eukaryotic communities in feces and xenic cultures of *Balantioides coli*. **A** Principal coordinates analysis (PCoA) based on Bray–Curtis dissimilarity, showing the distribution of fecal and culture samples obtained from family-based and industrial farms. Axes represent the percentage of explained variance. **B** Boxplots of within-group Bray–Curtis distances for fecal and culture samples. **C** Principal coordinates analysis (PCoA) based on Weighted UniFrac distance, showing the separation between fecal and culture samples while considering phylogenetic relationships among taxa. **D** Boxplots of within-group Weighted UniFrac distances for fecal and culture samples

Principal coordinates analysis showed differences in the unicellular eukaryotic community between *B. coli* xenic cultures and the corresponding fecal samples, with several *B. coli* ASVs detected at high abundance exclusively in cultures, whereas Entamoebidae ASVs were predominantly associated with fecal material (Fig. 3A, B). This pattern was supported by Bray–Curtis PERMANOVA, which indicated a trend toward differentiation between matrices (Pseudo-F = 2.26; p = 0.054). In contrast, the Weighted UniFrac analysis revealed a more subtle separation, and PERMANOVA showed no statistically significant difference (Pseudo-F = 1.72; p = 0.159) (Fig. 3C, D).

The composition of unicellular eukaryotes differed between pig production systems. Samples from family-based farms showed greater heterogeneity, with fluctuating dominance of *B. coli* and higher representation of *Blastocystis* spp. and Entamoebidae ASVs, whereas industrial farms displayed a more homogeneous profile, strongly dominated by *B. coli* (≥ 90%) with only occasional low-abundance detections of other taxa. Low-abundance ASVs of other protozoa with zoonotic potential, including *Cryptosporidium scrofarum* and *C. struthionis*, were also detected (Additional file 3: Table S3).

Despite these contrasting local patterns, global beta diversity analyses did not reveal statistically significant differences between production systems (Bray–Curtis: Pseudo-F = 0.343643; p = 0.922; Weighted UniFrac: Pseudo-F = 0.684081; p = 0.551), indicating overall similarity in both taxonomic composition and phylogenetic structure. However, samples from family-based farms showed greater dispersion, whereas those from industrial farms clustered more cohesively (Fig. 3B, D).

### Prokaryotic communities associated with *Balantioides coli*: comparison between fecal samples and xenic cultures, and between family-based and industrial farms

**Fig. 4:**
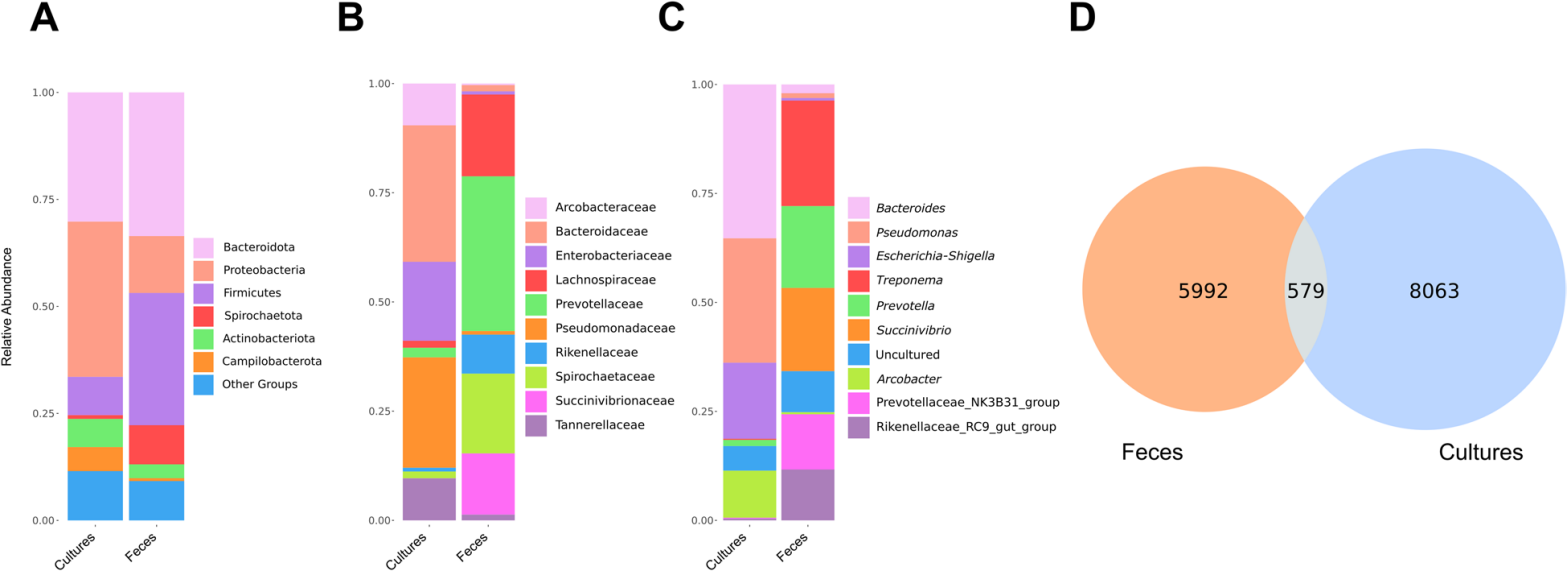
Comparative prokaryotic community structure between *Balantioides coli* xenic cultures and pig fecal samples by ASVs. The most abundant taxa are shown at the phylum (**A**), family (**B**), and genus (**C**) levels. The Venn diagram (**D**) illustrates the number of shared and exclusive ASVs between fecal samples and xenic cultures

The Venn diagram analysis of prokaryotic ASVs showed pronounced divergence between the profiles obtained from fecal samples and xenic cultures. Of the total ASVs detected, 5,992 were exclusive to fecal samples, whereas 8,063 were exclusive to xenic cultures containing *B. coli*. Only 579 ASVs were shared between both matrices (Fig. 4D).

Overall, the composition of the prokaryotic community varied substantially between pig feces and *B. coli* xenic cultures (Fig. 4A–C). Fecal samples showed greater representation of the phyla Bacteroidota and Firmicutes, followed by Proteobacteria, regardless of farm type. However, feces from family-based farms exhibited greater heterogeneity in the relative abundance of these phyla, whereas the profile from industrial farms was more homogeneous across individuals.

**Fig. 5:**
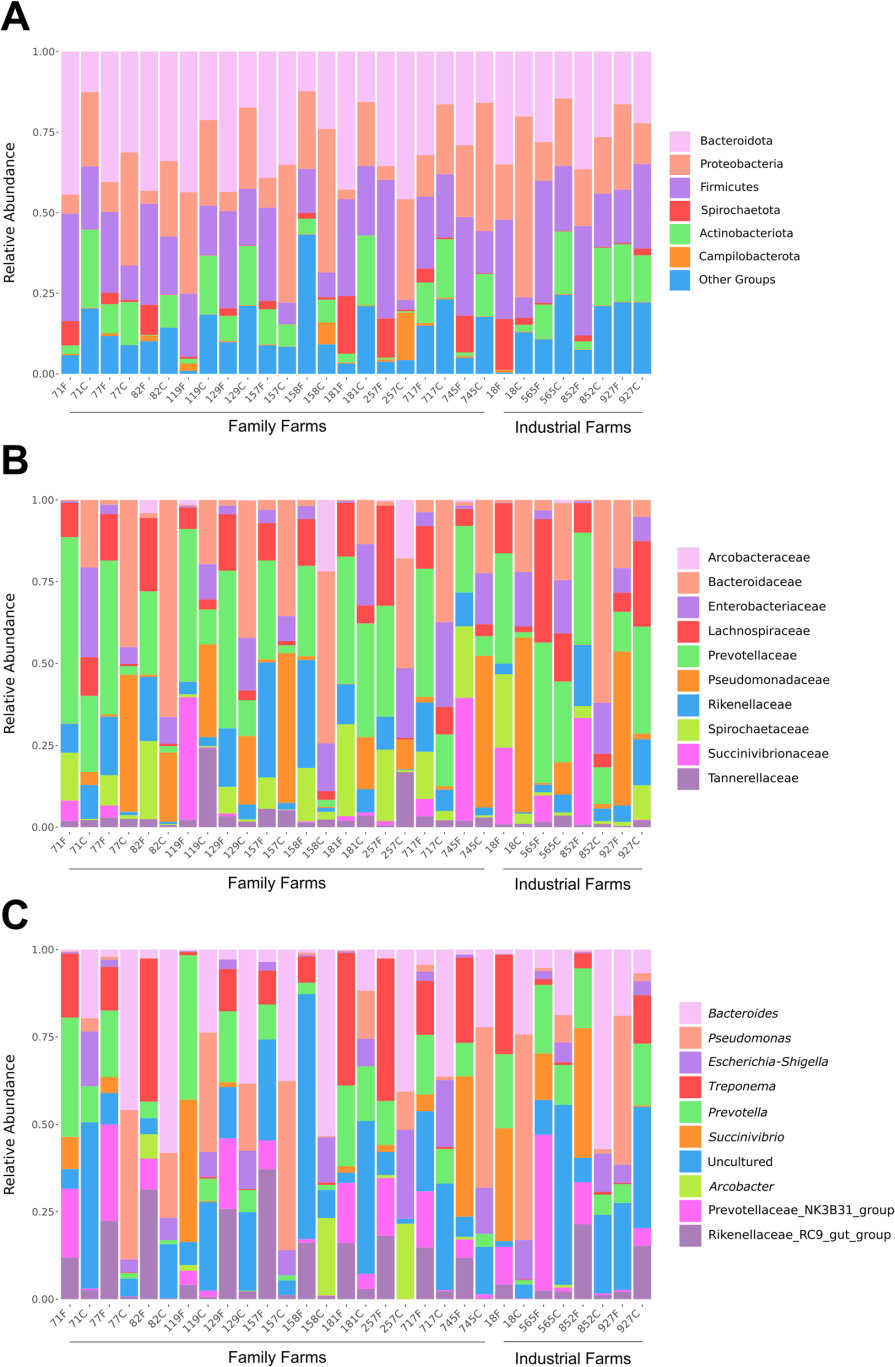
Pig farming system shapes prokaryotic ASVs in feces and *Balantioides coli* xenic cultures. Relative abundance of the most abundant prokaryotic phyla (**A**), families (**B**), and genera (**C**) in *Balantioides coli* xenic in vitro cultures and their corresponding pig fecal samples from family and industrial farms, shown individually for each sample

In contrast, *B. coli* xenic cultures displayed a more simplified community, characterized by a relative increase in Proteobacteria and Bacteroidota, together with a marked reduction in Firmicutes, a pattern consistently observed across both pig production systems (Figs. 4A and 5A).

At the family level, fecal samples were mainly dominated by Prevotellaceae, Lachnospiraceae, and Spirochaetaceae, with greater interindividual variability observed among family-based farms. In contrast, industrial farms showed a more homogeneous profile, particularly characterized by Prevotellaceae and Enterobacteriaceae, whose ASVs were detected in fecal samples from all animals included in the metabarcoding analyses.

In *B. coli* xenic cultures, a clear selection for specific families was observed, with a relative increase in Bacteroidaceae, Pseudomonadaceae, and Enterobacteriaceae (Figs. 4B and 5B).

Among the most abundant genera, fecal samples exhibited greater ASV taxonomic diversity, with predominance of *Prevotella*, *Treponema*, and *Succinivibrio.* These taxa were particularly frequent in feces obtained from family-based farms. By contrast, *B. coli* xenic cultures were characterized by a greater relative abundance of *Bacteroides*, *Pseudomonas*, and *Escherichia–Shigella* (Figs. 4C and 5C).

**Fig. 6:**
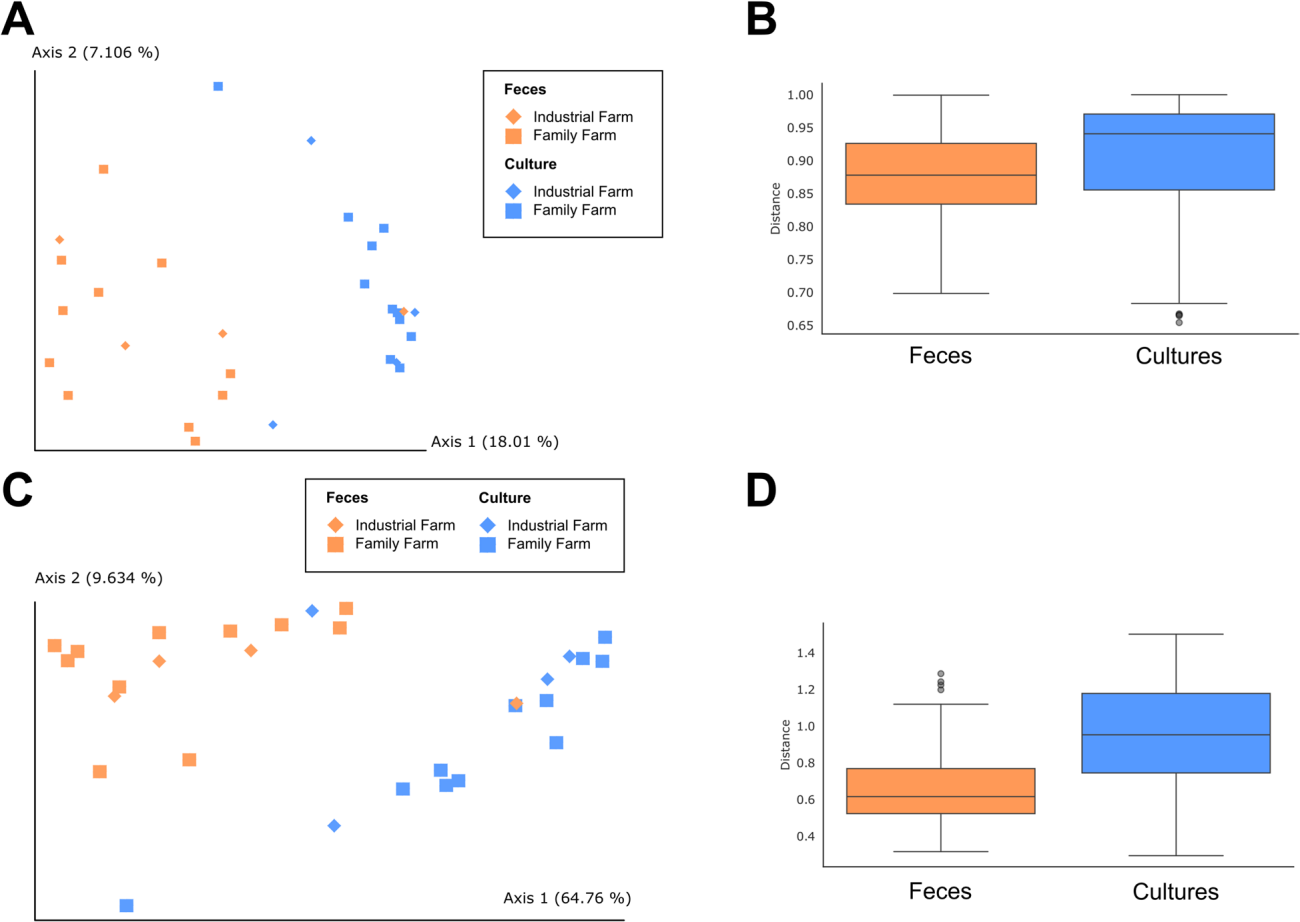
Prokaryotic compositional differences between feces and *Balantioides coli* cultures from family and industrial pigs. **A** Principal coordinates analysis (PCoA) based on Bray–Curtis distance, showing the distribution of fecal and culture samples obtained from family and industrial farms. The axes represent the percentage of variance explained. **B** Boxplots of within-group Bray–Curtis distances for fecal and culture samples. **C** Principal coordinates analysis (PCoA) based on Weighted UniFrac distance, highlighting the separation between fecal and culture samples while considering phylogenetic relationships among taxa. **D** Boxplots of within-group Weighted UniFrac distances for fecal and culture samples

Beta diversity analys**e**s revealed significant structural differences in the prokaryotic community between pig fecal samples and *B. coli* xenic cultures, irrespective of farm type, based on both Bray–Curtis and Weighted UniFrac distances (Fig. 6A–D). PCoA showed clear separation between fecal and culture samples, particularly in the Weighted UniFrac analysis. These visual patterns were statistically confirmed by PERMANOVA for both metrics (Bray–Curtis: Pseudo-F = 4.242; p = 0.001; Weighted UniFrac: Pseudo-F = 16.401; p = 0.001) (Fig. 6A–D).

In contrast, the comparison between samples obtained from family and industrial farms did not reveal statistically significant differences in prokaryotic community composition. Although some taxa showed higher relative frequencies in samples from specific farm types, PERMANOVA analysis did not indicate segregation between groups when all ASVs were considered, for either Bray–Curtis (Pseudo-F = 1.016; *p* = 0.373) or Weighted UniFrac (Pseudo-F = 1.228; *p* = 0.266).

### Spatial distribution of eukaryotic and prokaryotic communities associated with *Balantioides coli*-positive fecal samples

The mapping of pig farms with fecal samples positive for *B. coli*, based on metabarcoding data, revealed spatial heterogeneity in the composition of the associated eukaryotic and prokaryotic communities, both among municipalities and across different production systems (Additional file 4: Fig. S1). In family farms, greater variability was observed in the relative composition of eukaryotic taxa, with predominance of *B. coli* ASVs associated with different proportions of other intestinal protozoa, including representatives of *Blastocystis* spp., Entamoebidae, and other eukaryotes, in addition to variation in the associated bacterial composition. In contrast, industrial farms showed more homogeneous microbial community patterns in fecal samples positive for *B. coli*, with lower relative variation among the detected taxonomic groups (Additional file 4: Fig. S1).

Spatial analysis also revealed differences in bacterial community composition, with variation in the relative abundance of the main phyla across locations. Farms located in different municipalities exhibited specific bacterial signatures, suggesting the influence of local factors, such as management practices, environment, and production system, on the structure of the communities associated with *B. coli*-positive fecal samples (Additional file 4: Fig. S1).

### Correspondence analysis of cultured *Balantioides coli* ASVs and associated eukaryotic and prokaryotic communities

**Fig. 7:**
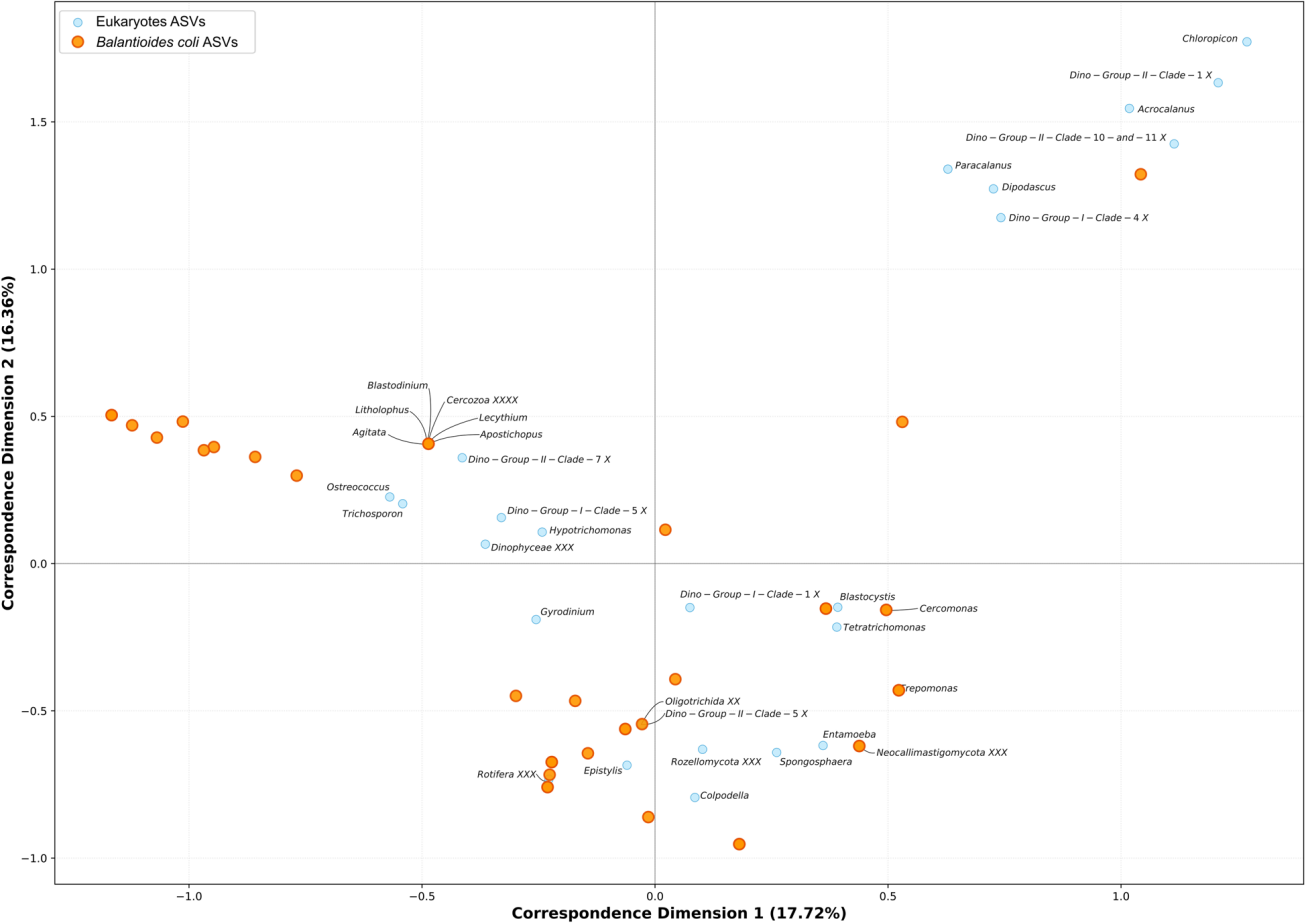
Correspondence analysis of *Balantioides coli* ASVs and associated eukaryotic taxa detected in xenic cultures. Orange points represent * Balantioides coli* ASVs, whereas blue points correspond to associated eukaryotic ASVs. The analysis was performed using a high-abundance cutoff (≥ 1000 ASVs) to highlight the taxa most strongly associated with * Balantioides coli* isolates maintained under in vitro culture conditions.

**Fig. 8:**
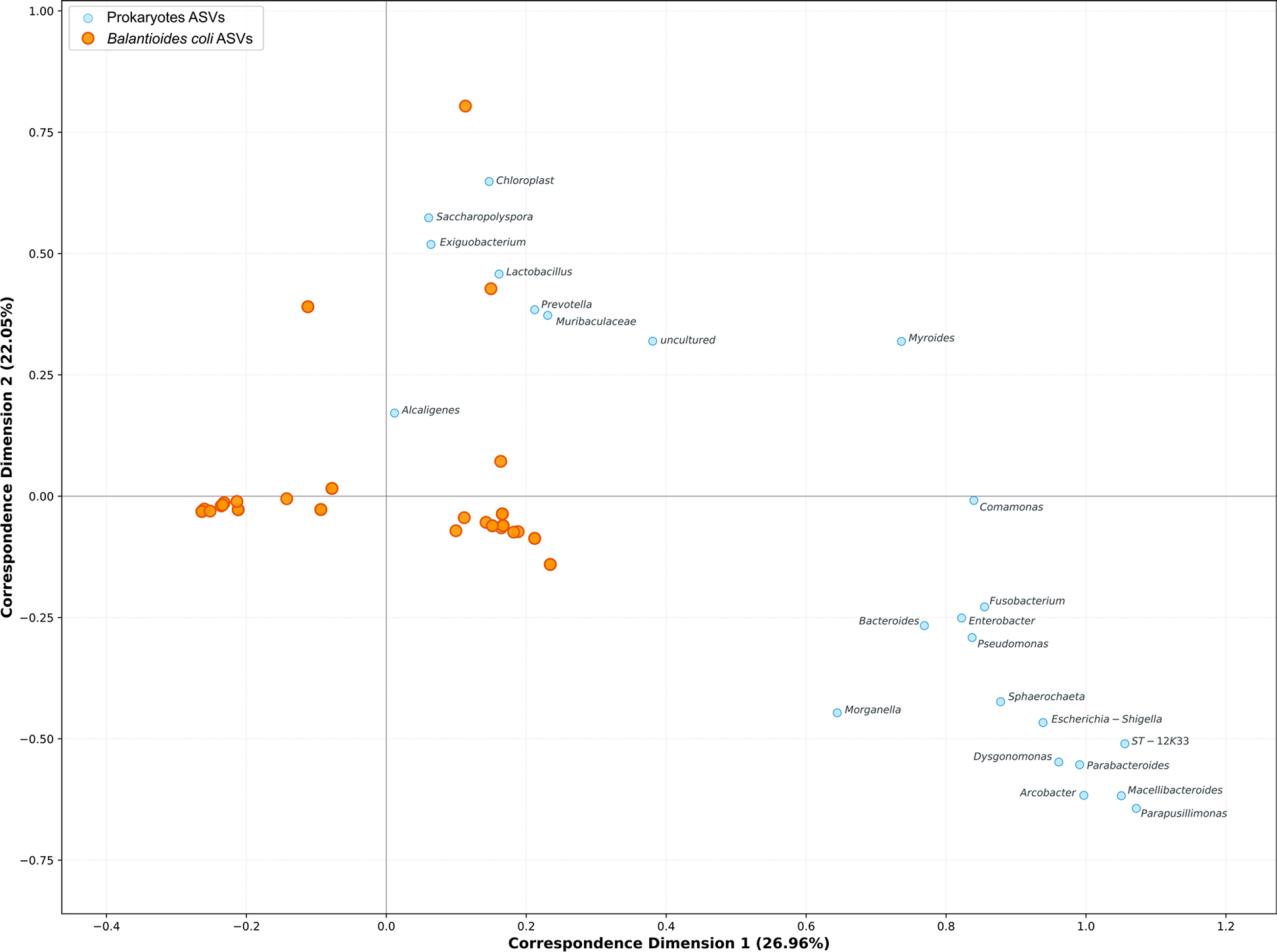
Correspondence analysis of *Balantioides coli* ASVs and associated prokaryotic taxa detected in xenic cultures. Orange points represent* Balantioides coli* ASVs, whereas blue points correspond to associated prokaryotic ASVs. The analysis was performed using a high-abundance cutoff (≥ 1000 ASVs) to highlight the taxa most strongly associated with *Balantioides coli* isolates maintained under in vitro culture conditions.

Correspondence analysis showed that the first two dimensions explained 34.08% of the total variability in the eukaryotic dataset and 49.01% in the prokaryotic dataset (Figs. 7 and 8).

*Balantioides coli* ASVs exhibited heterogeneous distribution patterns and variable associations with surrounding eukaryotic taxa (Fig. 7). In the eukaryotic dataset, distinct *B. coli* ASVs were positioned near representatives of Dinophyceae (Dino Group I and II), fungi (*Dipodascus*), copepods (*Acrocalanus* and *Paracalanus*), and protists such as *Blastocystis*, *Cercomonas*, *Tetratrichomonas*, and *Entamoeba*. Additional associations were observed with environmental and free-living taxa, including *Blastodinium*, *Litholophus*, *Apostichopus*, *Lecythium*, and representatives of Cercozoa (Fig. 7).

Correspondence analysis of the prokaryotic dataset revealed the formation of three major co-occurrence structures involving *B. coli* ASVs and bacterial taxa within xenic cultures. One large cluster comprised multiple *B. coli* ASVs positioned in close linear association with bacterial genera such as *Bacteroides*, *Pseudomonas*, *Escherichia–Shigella*, and *Enterobacter*. In contrast, a second prominent group of *B. coli* ASVs occupied relatively isolated positions in the ordination space and did not cluster closely with dominant bacterial taxa, considering the established cutoff threshold of at least 1000 ASVs. Additionally, a third and smaller cluster composed of fewer *B. coli* ASVs was positioned near bacteria including Firmicutes (*Lactobacillus* and *Exiguobacterium*), Bacteriodota (*Prevotella* and *Muribaculaceae*) and Actinomycota (*Saccharopolyspora*) (Fig. 8).

## Discussion

Overall, structures morphologically compatible with *B. coli* were detected by microscopy and successfully isolated in xenic culture from fecal samples obtained from all farms included in this study. Priority was given to samples with higher numbers of compatible forms, including viable trophozoites, as recommended in previous studies [20, 21, 44]. Nevertheless, successful isolation was achieved from one fecal sample that tested negative by microscopy after blind inoculation into culture medium immediately following microscopic examination. Direct microscopic examination is widely used for the detection of intestinal parasites such as *B. coli*; however, only a small fraction of the fecal material is examined, which may limit detection in low-intensity infections. After the fifth day of incubation, the cultured isolate was confirmed as positive for *B. coli* by PCR, and molecular analysis also confirmed the presence of the parasite in the original fecal sample, demonstrating that the negative microscopic result likely reflected the limited sensitivity of direct examination. These findings reinforce the value of complementary molecular approaches and support blind inoculation as a useful strategy for parasite recovery, particularly in preferred hosts such as pigs.

Most isolates remained viable in vitro for short periods, generally between two and four weeks, consistent with previous reports [20, 21, 45, 46]. Even so, some isolates showed prolonged viability, suggesting that both isolate-specific characteristics and culture conditions may influence persistence. Given the absence of consolidated cryopreservation protocols for *B. coli* [2], in vitro maintenance remains labor-intensive and requires frequent subculturing, which should be considered when designing experimental studies involving this protozoan.

Molecular and phylogenetic analyses confirmed the presence of *B. coli* in all fecal samples and corresponding cultured isolates, with clear segregation of sequences into the A0 and B0 clades and high topological support. The sequences generated in this study clustered with reference isolates previously reported from Europe, Africa, Asia, and the Americas [12, 14, 15, 18, 20, 27, 47–50], reinforcing the genetic conservation and broad geographic distribution of these lineages. The predominance of the B0 variant in both fecal samples and cultures is consistent with previous reports indicating that this is the most widely distributed lineage in pig populations [19, 20], whereas A0 was detected at a lower frequency and showed a more restricted distribution.

Although the ecological adaptive advantages of the B0 variant remain unclear, its predominance in both fecal samples and xenic cultures in the present study may suggest greater adaptation to pig hosts and/or higher tolerance to in vitro conditions. From a One Health perspective, this finding deserves attention because pigs are recognized as the main reservoirs of *B. coli* and maintain close contact with humans in rural production systems. Supporting this hypothesis, our research group recently detected the A0, B0, and atypical variants of *B. coli* in fecal samples from pig farmers occupationally exposed to infected pigs in Brazil, reinforcing the zoonotic potential and possible interspecies transmission of these lineages at the animal–human interface [2].

The analysis of matched feces–culture pairs allowed direct assessment of lineage stability after in vitro establishment. In most cases, fecal and culture-derived sequences clustered within the same phylogenetic subclade and showed minimal within-host genetic distances, indicating high genetic concordance and suggesting that the culture process largely preserved the original genetic identity of the variants. However, specific exceptions were observed in some feces–culture pairs, in which the genetic variant detected after in vitro establishment differed from that identified directly in the original fecal sample. This pattern suggests that xenic culture may favor the differential expansion of pre-existing parasitic subpopulations, acting as a selective environment for variants with greater adaptation to in vitro conditions. Similar observations were previously reported for *B. coli* isolates maintained in xenic culture systems differing only in serum composition and originating from the same pig fecal sample [20].

In addition, fecal samples may contain genetically heterogeneous populations of *B. coli*, and mixed infections cannot be excluded. Likewise, intragenomic polymorphism of rRNA genes should also be considered, given the coexistence of at least two ribosomal gene types previously described in the *B. coli* genome [18, 51]. Conjugation events in ciliates should also be considered, as they may contribute to genetic recombination and sequence variation after culture establishment. Although no double peaks were observed in the electropherograms, preferential amplification of the most abundant variant cannot be excluded. Additional approaches such as variant-specific qPCR, cloning, or deep sequencing will be necessary to confirm mixed infections and better understand the dynamics of *B. coli* variants during in vitro establishment.

Metabarcoding analysis comparing the eukaryotic and prokaryotic communities present in *B. coli* xenic in vitro cultures and in the corresponding pig fecal samples showed that parasite isolation and maintenance promoted a reconfiguration of the unicellular microeukaryotic community originally present in feces. Although part of the ASVs was shared between both matrices, the high proportion of culture-exclusive ASVs indicates that the in vitro environment acts as an ecological filter, selecting organisms capable of surviving and proliferating under the culture conditions.

In this context, *B. coli* and *Blastocystis* spp. were the most frequently detected eukaryotes in cultures, which was expected given their routine microscopic detection during subculture procedures and the recognized suitability of the medium for intestinal protozoa [12, 20, 21, 52, 53]. The culture system also favored the multiplication of these organisms, resulting in their predominance within the isolates. In contrast, fecal samples exhibited greater eukaryotic diversity, particularly of representatives of the family Entamoebidae. The identification of *E. suis*, *E. polecki*, *E. moshkovskii*, and *E. hartmanni* highlights the complexity of the pig intestinal microeukaryotic community. The lower recovery of these taxa in culture may be related to the scarcity of established in vitro protocols for most *Entamoeba* species, unlike what is available for *E. histolytica* and *E. moshkovskii* [44, 54].

The present study was not primarily designed to investigate the epidemiology of *Entamoeba* spp. in pigs; nevertheless, these findings are highly relevant, as they represent the first molecular evidence of Amoebozoa members in pig feces in Brazil. Similar observations have been reported in weaned pigs from Denmark [55]. From an epidemiological perspective, this detection deserves attention because some species, such as *E. polecki* and *E. moshkovskii*, have recognized zoonotic potential and may represent a risk at the animal–human interface [56–58]. In addition, evidence suggests that *E. suis* and *E. polecki* may also be associated with clinically relevant intestinal lesions in pigs, including mucosal invasion and hemorrhagic colitis [59–61].

Overall, culture reduced part of the original diversity of ASVs from other protozoa; however, *Blastocystis* spp. and members of Entamoebidae remained detectable in some isolates. This pattern suggests that certain intestinal eukaryotes exhibit greater tolerance to xenic conditions and may persist alongside *B. coli* in culture, possibly by exploiting similar metabolic niches or benefiting from bacterial activity within the medium. Correspondence analyses further supported this interpretation, as some—but not all—*B. coli* ASVs clustered near intestinal protists such as *Blastocystis*, *Entamoeba*, and members of Parabasalia, indicating recurrent co-occurrence patterns among intestinal eukaryotes within xenic cultures. However, the scattered distribution and relatively isolated positioning of several *B. coli* ASVs suggest that the ciliate does not depend on specific eukaryotic taxa for persistence in vitro, despite its ability to coexist with diverse protists and environmental eukaryotes within the same culture system.

Marked proliferation of other protozoa in the culture medium, particularly *Blastocystis* spp., was observed in some isolates and frequently compromised the viability of *B. coli*, requiring successive and labor-intensive subcultures to maintain viable isolates. Similar difficulties have previously been reported in xenic cultures involving *Entamoeba histolytica*/*E. dispar*, in which *Blastocystis* interfered with culture stability [20, 62–64]. Frequent subculturing may therefore have contributed to maintaining these mixed eukaryotic communities while minimizing the deleterious effects of protozoan overgrowth on ciliate viability.

Beyond its methodological impact, the recurrent detection of *Blastocystis* spp. is also epidemiologically relevant. This protozoan is widely distributed in pigs and has recognized zoonotic potential [65–67]. Although its pathogenic role in humans remains controversial [68, 69], blastocystosis remains poorly explored in veterinary parasitology, with limited information regarding its epidemiology and possible implications for animal health.

The separation between fecal samples and cultures observed in the Bray–Curtis-based PCoA indicates that culture substantially altered the relative abundance of the eukaryotic community. In contrast, the less pronounced separation in the Weighted UniFrac analysis suggests that the main phylogenetic groups, including *B. coli*, *Blastocystis* spp., and *Entamoeba* spp., remained partially shared between matrices, despite changes in abundance. Thus, culture appears to restructure the community rather than introduce entirely new protozoan clades.

Additionally, the detection of other parasites, such as *Cryptosporidium scrofarum* and *C. struthionis*, even at low ASV abundance, reinforces the epidemiological relevance of the pig intestinal protist community. However, it is important to note that NGS-based approaches still present limitations for precise species-level identification of *Cryptosporidium* [55]. In Brazil, *C. scrofarum* has already been reported in domestic and feral pigs [70, 71], whereas the detection of *C. struthionis* represents an unusual finding, previously described in cattle feces in China [72]. In the present study, its occurrence was restricted to family farms (C and K), suggesting possible environmental contamination by bird feces or other host species, potentially associated with less stringent sanitary management.

The structure of the prokaryotic communities associated with *B. coli* showed marked differences between pig feces and xenic cultures. This selective pattern was expected, as xenic cultures naturally favor microorganisms capable of persisting and multiplying under artificial in vitro conditions. However, to our knowledge, no previous studies have directly compared the microbiota of intestinal protozoan xenic cultures with that of the original fecal samples. Thus, the present findings provide a novel contribution to understanding the interactions between *B. coli* and associated bacterial communities under culture conditions.

The prokaryotic community detected in culture did not fully reflect the original intestinal microbiota, but rather a functionally adapted subset of microorganisms suited to the in vitro environment, potentially involved in protozoan maintenance, nutrition, or metabolism. This interpretation is supported by previous observations of bacteria within food vacuoles in the cytoplasm of *B. coli*, suggesting their possible role as a nutritional source or metabolic partner [73].

Beta diversity analyses reinforced these structural differences, showing consistent separation between fecal and culture matrices in both Bray–Curtis and Weighted UniFrac metrics. The higher Pseudo-F value observed for Weighted UniFrac indicates that xenic culture altered not only the relative abundance of bacterial taxa but also the phylogenetic composition of the community, suggesting selection of lineages evolutionarily distinct from those predominant in the original intestinal environment.

At a broad taxonomic level, fecal samples were dominated by Firmicutes and Bacteroidota, followed by Proteobacteria, consistent with the classical gut microbiota profile previously reported in pigs infected with *B. coli* [74, 75]. Among the most abundant genera, fecal samples showed greater taxonomic diversity, with predominance of *Prevotella*, *Treponema*, and *Succinivibrio*. These genera are involved in the production of metabolites involved in host energy metabolism and intestinal homeostasis [76–81]. In pigs, *Prevotella* has been linked to both beneficial productive traits and diarrheic conditions, particularly in animals coinfected with enteric pathogens, including *B. coli* [74–76]. Despite the recognized ecological importance of these genera, their potential association with *B. coli* remains poorly understood, highlighting an important gap in the study of protozoan–bacterial interactions in pigs.

In contrast, xenic cultures showed lower taxonomic diversity, with enrichment of Proteobacteria and Bacteroidota. The predominance of families such as Bacteroidaceae, Pseudomonadaceae, and Enterobacteriaceae, together with genera including *Bacteroides*, *Pseudomonas*, *Escherichia*–*Shigella*, and *Enterobacter* suggests selective growth of metabolically versatile bacteria able to rapidly adapt to the culture medium. The marked presence of these bacterial taxa in *B. coli* xenic cultures was further supported by correspondence analyses, in which multiple ASVs of the ciliate co-occurred with prokaryotes belonging to this group under in vitro conditions, highlighting not only their overall abundance but also their predominance across different ciliate isolates.

*Bacteroides* may support protozoan maintenance through the production of short-chain fatty acids and other fermentation metabolites that help sustain a low-oxygen microenvironment compatible with the facultatively anaerobic physiology of *B. coli* [74]. The high abundance of *Pseudomonas* likely reflects its metabolic versatility and competitive advantage in Pavlova medium, consistent with the bluish–greenish coloration observed in some cultures [21, 82, 83]. Likewise, the persistence of *Escherichia–Shigella* probably reflects successful establishment of enterobacteria originating from fecal material, preserving microbial interactions relevant to protozoan physiology [44].

Furthermore, the spatial analysis of pig farms, integrating both geographic location and production system, revealed heterogeneity in the composition of eukaryotic and prokaryotic communities associated with fecal samples positive for *B. coli* ASVs, in agreement with previous findings reporting greater parasite diversity in family-based systems [84]. However, despite local differences in taxon abundance and spatial variability, no statistically significant overall segregation was observed between production models, indicating that the broader microbial community structure remains largely shared across systems. This pattern likely reflects the recurrent detection of *B. coli* across all farm types and suggests that, although stricter sanitary management may reduce the circulation of specific microorganisms, it may be insufficient to interrupt the transmission of *B. coli* and other taxa potentially involved in its intestinal colonization and persistence.

Due to the high costs of large-scale sequencing, one representative sample per farm was selected for cultivation and metabarcoding analyses. Although this approach enabled the inclusion of all farms, the non-random sampling design may have underestimated within-farm microbial variability and limits broader generalization of the observed patterns. Likewise, comparisons between family-based and industrial farms should be interpreted with caution, as the sampling design did not allow a comprehensive assessment of potentially associated factors, including stocking density, diet, biosecurity practices, and medication history. Therefore, the differences observed between production systems remain exploratory and highlight the need for future studies using expanded and pooled sampling strategies to better disentangle how management practices and environmental variables may shape microbial communities potentially associated with *B. coli* colonization.

## Conclusions

Taken together, our findings show that *B. coli* circulates widely in pigs from distinct production systems in southeastern Brazil and can be successfully maintained in xenic culture using fecal samples from naturally infected animals. Although the parasite remained relatively genetically stable after in vitro establishment, culture acted as a selective filter for both parasite variants and the associated microbiota. These results indicate that *B. coli* should be interpreted as part of a complex intestinal microbial consortium rather than as an isolated parasite, reinforcing the need for an integrative ecological approach to better understand its transmission, adaptation, and zoonotic relevance.

## Supplementary Information

Additional file 1. Table S1 Fecal samples and cultured isolates by municipality and farm for sequencing analyses in southeastern Brazil. A–O correspond to the pig farms included in the study. Values are expressed as the number of fecal samples and corresponding cultured isolates analyzed

Additional file 2. Table S2. Distance matrix showing the estimated values of evolutionary divergence between sequences, expressed as the number of base substitutions per site. Values represent pairwise genetic distances among sequences. Sequence identifiers correspond to sample IDs

Additional file 3. Table S3 Amplicon sequence variantsdetected in fecal and *in vitro* culture samples, with read counts per sample. Columns represent sample IDs, and rows correspond to unique Feature IDs. Taxonomic classification is provided from domain to species level. “Confidence” indicates the taxonomic assignment score

Additional file 4. Fig. S1. Spatial distribution of eukaryotic and prokaryotic communities in *Balantioides coli*-positive pig feces. Spatial distribution of eukaryoticand prokaryoticcommunities associated with *Balantioides coli*-positive fecal samples obtained from family and industrial pig farms in the states of Rio de Janeiro and Minas Gerais, Brazil. Letters A to K indicate family farms located in Maricá, Cachoeiras de Macacu, Silva Jardim, Rio Bonito, Itaboraí, Saquarema, Casimiro de Abreu, and Tanguá, whereas industrial farms are represented by Pinheiral, Nova Friburgo, Rio Pomba, and Barbacena. Pie charts represent the relative composition of the main eukaryoticand bacterialgroups detected by metabarcoding in *B. coli*-positive fecal samples.

## Data Availability

All data generated or analyzed during this study are included in this published article and its supplementary information files.
