# Supplementary figures and images for: From farm to flask: stable genotypes and shifting microbiomes in the ecological dynamics of Balantioides coli from a One Health perspective
Source: Parasit Vectors. 2026 Jul 7;19:289. doi: 10.1186/s13071-026-07513-y (PMC13366902; doi:10.1186/s13071-026-07513-y)

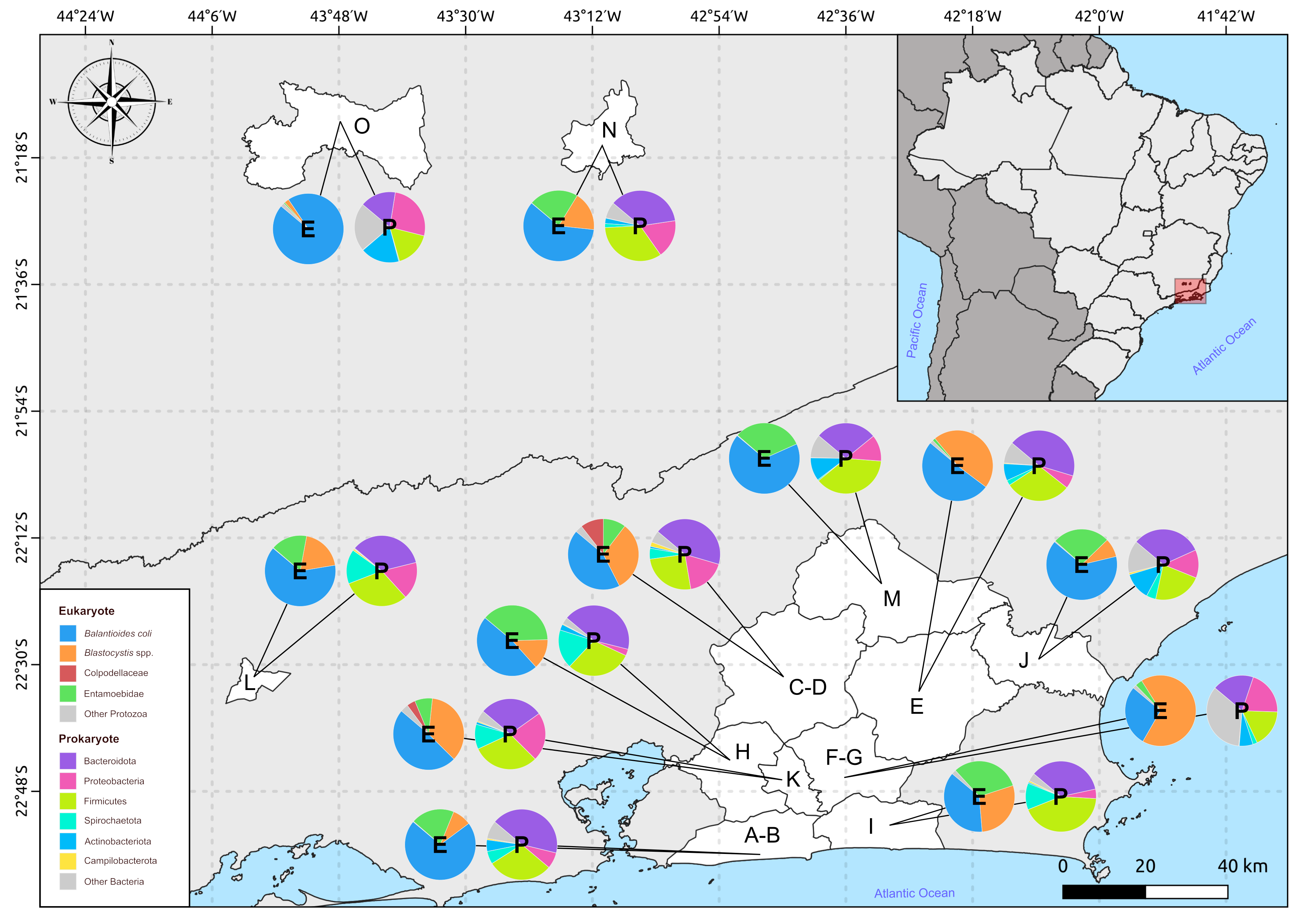

Supplement: Supplementary file 4 — Additional file 4. Fig. S1. Spatial distribution of eukaryotic and prokaryotic communities in Balantioides coli-positive pig feces. Spatial distribution of eukaryoticand prokaryoticcommunities associated with Balantioides coli-positive fecal samples obtained from family and industrial pig farms in the states of Rio de Janeiro and Minas Gerais, Brazil. Letters A to K indicate family farms located in Maricá, Cachoeiras de Macacu, Silva Jardim, Rio Bonito, Itaboraí, Saquarema, Casimiro de Abreu, and Tanguá, whereas industrial farms are represented by Pinheiral, Nova Friburgo, Rio Pomba, and Barbacena. Pie charts represent the relative composition of the main eukaryoticand bacterialgroups detected by metabarcoding in B. coli-positive fecal samples. [file 13071_2026_7513_MOESM4_ESM.tiff]
